# The Effect of Injection Molding Temperature on the Morphology and Mechanical Properties of PP/PET Blends and Microfibrillar Composites

**DOI:** 10.3390/polym8100355

**Published:** 2016-10-09

**Authors:** Maja Kuzmanović, Laurens Delva, Ludwig Cardon, Kim Ragaert

**Affiliations:** Research Group Center for Polymer and Material Technologies, Department of Materials Science & Engineering, Faculty of Engineering and Architecture, Ghent University, Technologiepark 915, 9052 Zwijnaarde, Ghent, Belgium; Maja.Kuzmanovic@UGent.be (M.K.); Laurens.Delva@UGent.be (L.D.); Ludwig.Cardon@UGent.be (L.C.)

**Keywords:** microfibrillar composites, injection molding, thermal properties, morphology, mechanical properties

## Abstract

Within this research the effect of injection molding temperature on polypropylene (PP)/poly(ethylene terephthalate) (PET) blends and microfibrillar composites was investigated. Injection molding blends (IMBs) and microfibrillar composites (MFCs) of PP/PET have been prepared in a weight ratio 70/30. The samples were processed at three different injection molding temperatures (*T*_im_) (210, 230, 280 °C) and subjected to extensive characterization. The observations from the fracture surfaces of MFCs showed that PET fibers can be achieved by three step processing. The results indicated that *T*_im_ has a big influence on morphology of IMBs and MFCs. With increasing the *T*_im_, distinctive variations in particle and fiber diameters were noticed. The differences in mechanical performances were obtained by flexural and impact tests. Establishing relationships between the processing parameters, properties, and morphology of composites is of key importance for the valorization of MFC polymers.

## 1. Introduction

Polyolefins are a class of polymers which are quite inexpensive and very easy to process. However, these polyolefins, such as polypropylene (PP) and polyethylene (PE), are often blended with engineering polymers, such as poly(ethylene terephthalate) (PET), polyamide (PA), or polycarbonate (PC) [1,2,3,4,5], which have superior mechanical and thermal properties. By combining both the easy processability of polyolefins and the high properties of engineering polymers a product could be obtained, which satisfies all performance demands [5]. Polypropylene (PP) is a semicrystalline polymer which represents one of the most commonly used polymeric materials. As a cheap polymer, its composites are of great interest, i.e., blends with PET which can improve dyeability and mechanical properties [6,7,8]. Poly(ethylene terephthalate) (PET) is a semicrystalline thermoplastic polymer with high toughness, and good physical and chemical properties. This set of properties makes PET of huge importance to the packaging industry. The main applications of this polymer are the manufacturing of films, fibers, and in the fabrication of bottles for beverages [6,7,9,10,11,12].

Researchers consider the blending of polymers as an economical and practical method to obtain new materials with adjusted properties, but very frequently different polymers are immiscible in the melt phase [3,13,14,15,16,17,18]. This immiscibility of polymers causes phase separation and low adhesion between two phases [4], which results in low mechanical properties [6,19]. Today, studying the phase separation of polymer blends is of great interest in determining the performance of new polymeric materials [20].

**Figure 1:**
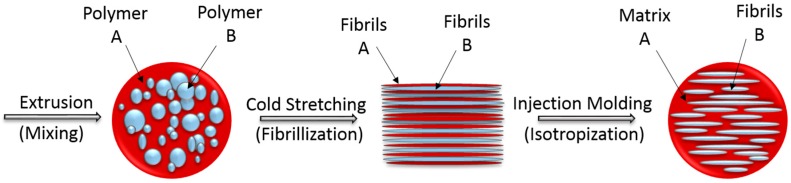
Scheme of MFC preparation [27,28].

Two different approaches are often employed to avoid the drawbacks of this immiscibility. The first one consists in the addition of a compatibilizer to these blends. Mostly copolymers are used which can interact with both polymeric building components. When it is added to the mixtures, it will result in smaller dispersion of the second phase and better adhesion between the phases. Excellent mechanical properties of the materials could be achieved. In some cases, the compatibilized blends have obtained the tensile fracture behavior from brittle to ductile [21]. A second option is to transform the blends into microfibrillar composites (MFCs). MFC are an interesting class of composites where a high melting fibrillated polymer reinforces a lower melting matrix polymer by applying a specific processing sequence. They could be utilized in a wide range of applications because of improved mechanical properties such as strength and stiffness [12,15,16,22]. The manufacturing of MFCs consists of three basic steps (Figure 1):

1. Extrusion–melt blending of the two immiscible polymers which have different melting temperatures *T*_m_ (mixing);
2. Hot or cold stretching of the extrudate with a molecular orientation of the two polymers (fibrillation);
3. Injection or compression molding–forming treatment at processing temperature of the lower melting component (isotropization) [3,14,23,24].

Fakirov et al. [22,25] and Evstatiev et al. [26] introduced the concept of microfibrillar composites (MFC) from polymer blends many years ago. These researchers stated that the processing of immiscible polymers in which the dispersed phase forms in situ reinforced fibers is a good way to achieve high properties [3]. Two main factors determine the improvement of mechanical properties: (i) the adhesion between matrix and reinforcement; and (ii) the aspect ratios (length/thickness ratio) of the reinforcing elements [19].

An interesting study by Friedrich et al. [27] hypothesized that, within MFCs, a copolymeric interface can be formed, which could play the role of a self-compatibilizer, meaning that by merely introducing this concept, the composites could already have better properties. This is possible in the case of condensation polymers. The improvement of mechanical properties by the MFC concept has already been shown [3,4,14], although not much attention has been dedicated to the specific influence of the processing temperature during the injection molding step (isotropization).

However, similar research for fiber-reinforced polymeric systems has demonstrated the importance of the injection molding temperatures on the properties of these composites. For natural fibers, this temperature strongly influences the fiber length, fiber diameter and, hence, the final mechanical properties [29]. For the MFC technique, most research focuses on differences in composition [14], influence of draw ratio [13,30], and influence of compatibilizers [15,17,21,28]. Several researchers [13,31] found that the influence of screw speed, barrel temperature, cooling time during molding, and mold temperature are very important for the final material properties.

No studies, however, extensively reported and explained as a main topic the effect of processing parameters on morphology and mechanical properties of PP/PET composition with the focus on the injection molding temperature.

The goal of this research is to investigate the influence of the injection molding temperature on the properties of the (non-stretched) injection molding blends (IMBs) and microfibrillar composites (MFCs), which are expected to originate within the morphology of the PET phase in PP/PET blends and composites. The IMBs were prepared by extrusion and injection molding while, for the preparation of MFCs, an additional cold stretching step was introduced between extrusion and injection molding. The relevant mechanical and thermal properties were investigated by flexural and impact testing and differential scanning calorimetry (DSC), respectively. The thermal degradation of the blends and composites was studied by thermogravimetric analysis (TGA). Further, scanning electron microscopy (SEM) was used to investigate the morphology of the samples and study the influence of different injection molding temperatures.

In this study, we present the results of non-compatibilized blends and composites as we would like to explain how, specifically, the injection molding temperature (*T*_im_) affects the main morphology and mechanical properties made from two virgin polymers, i.e., without additives, compatibilizers, or fillers. The main contribution of this work is that it gives a better insight into the temperature-based morphological development of MFCs during manufacturing. This fundamental insight remains relevant when expanding research towards the incorporation of compatibilizing agents.

## 2. Materials and Methods

### 2.1. Materials

The polypropylene (PP) was purchased from Sabic (Sabic 575P, Bergen op Zoom, The Netherlands) with a melt flow rate (MFR) of 11 g/10 min (2.16 kg, 230 °C), and the used polyethylene terephthalate (PET) was LIGHTER C93 (Equipolymers, Schkopau, Germany), which is a bottle-grade material with an intrinsic viscosity of 0.80 ± 0.02 dL/g. PET was dried in a vacuum oven for 24 h at 60 °C before processing, while PP was used as received.

### 2.2. Preparation of PP/PET (Polypropylene/Polyethylene Terephthalate), IMBs (Injection Molding Blends) and MFCs (Microfibrillar Composites)

**Figure 2:**
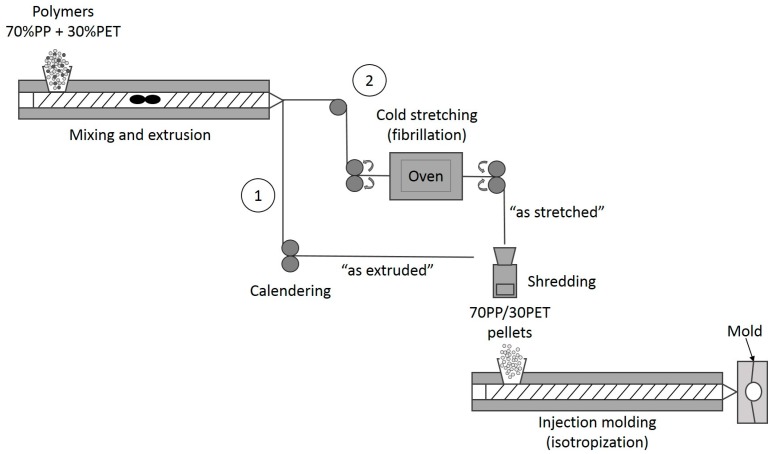
Processing scheme of: (**1**) IMBs (Injection molding blends) and (**2**) MFCs (Microfibrillar Composites).

In this work, PP was used as a matrix, and PET as a reinforcing element. Materials were prepared in a weight ratio of 70/30 PP/PET. The sample preparation was divided into IMB and MFC preparation, respectively. The IMB samples were prepared in two basic steps (Figure 2, processing route 1): Extrusion and injection molding. The PP and dried PET pellets were properly dry-mixed in a fixed weight ratio of 70/30 before being added to the hopper. The first step, melt blending, was done at the processing temperature of the component with the higher *T*_m_ using a twin-screw extruder (Coperion ZSK18, Stuttgart, Germany) with a die opening of 19 mm × 2 mm. The screw speed was set at 120 rpm and the barrel temperatures were placed from 205 to 260 °C. The extrudate has been obtained as a sheet with dimensions of 25 mm × 1 mm, by passing through calander rolls, which were cooled down to ~15 °C. Further on, the sheets were shredded and injection molded with a BOY 22S injection molding machine (BOY, Neustadt-Fernthal, Germany). In order to study the effect of injection molding temperature, samples were injection molded at three different *T*_im_: 210, 230, and 280 °C.

The manufacturing of MFCs consists of three steps (Figure 2, processing route 2). The blending extrusion temperatures for both IMB and MFC were maintained equal. The first step is the same: melt blending of PP and PET by extrusion. Then the received cooled extrudate was entered directly into a hot oven (200 °C, 55.5 cm × 60 cm) and stretched above the glass transition temperature of PET at a measured surface temperature of 95 °C by a pair of rolls. The speed of the rolls was adjusted to obtain a draw ratio of four. This step is called fibrillation.

Before the final isotropization step, the stretched blend was granulated by using a shredder (Piovan RSP15/30, Piovan, Maria di Sala, Italy), and processed by injection molding at the same temperatures as the IMBs. The mold consisted of rectangular bars with dimensions of 100 mm × 10 mm × 4 mm, suited for both impact testing and three-point bending.

### 2.3. Characterization of PP/PET IMBs and MFCs

Thermogravimetric analysis of the virgin polymers and composites was performed on a STA449 Netzsch device (Selb, Germany) in the temperature interval from 30–600 °C with a heating rate of 10 and 20 mL·min^−1^ flow of nitrogen gas. The onset degradation temperature was calculated at 5% of mass loss for all samples. Differential scanning calorimetry (DSC) was employed to investigate the crystallization and melting behavior. Measurements were performed in two cycles of heating-cooling in the temperature range between 20–300 °C by a Netzsch DSC 204F1 device (Selb, Germany) under nitrogen atmosphere, the heating/cooling rate was 10 °C·min^−1^, and the flow of nitrogen gas was 20 mL·min^−1^. α_c_ was calculated for the PP phase based on the theoretical enthalpy for 100% crystalline polymer and taking the mass percentage into account (Equation (1)):

(1) $$\alpha_{c}=\frac{\Delta H^{exp}}{\Delta H˚\;w_{f}}\;\cdot 100\%$$

where **Δ*H***^o^ for PP is 207 J/g [32], and ***w_f_*** is the weight fraction of the relevant polymer in the PP/PET composition.

To study the morphology of IMBs and MFCs, the specimens were prepared for microscopy by immersing in liquid nitrogen and, consequently, fracturing them. Then, the samples were sputtered with gold by a Bal-Tec SCD005 sputter coater (Bal-Tec, Balzers, Liechtenstein). Micrographs were obtained by scanning electron microscopy (SEM), more specifically, a FEG SEM JEOL JSM-7600F 202 instrument (Tokyo, Japan). The accelerating voltage was 15 kV. The average diameter of the particles and fibrils was determined by Image J software (National Institutes of Health, Bethesda, MD, USA). For the calculation, at least 30 measurements were used.

The three-point bending flexural test was done with an Instron 3601 testing machine (Norwood, MA, USA) with a load cell of 2 kN according to standard ISO 178. The sample was placed on two supporting spans with a set distance of 64 mm apart and, from above, a third loading pin was lowered at a constant rate (5 mm·min^−1^) until sample failure.

Charpy impact strength was measured using a Tinius Olsen IT 503 Pendulum Impact Tester machine (Ulm, Germany) according to standard ISO 179. The specimens were notched at 2 mm depth and broken by a hammer with an energy value of 2 J. At least five specimens were tested for both flexural and impact tests. Statistical analysis of all results was performed by software package SPSS Statistics 22 (Armonk, NY, USA) (*t*-independent sample tests, *p* = 0.05).

## 3. Results

### 3.1. Thermal Properties of IMBs and MFCs

**Figure 3:**
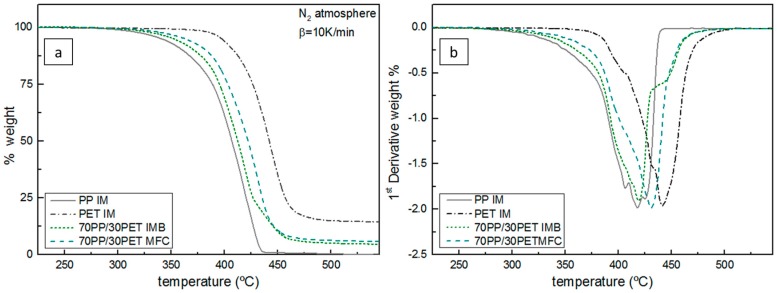
Dynamic (**a**) TG curves and (**b**) dTG of PP IM, PET IM, 70PP/30PET IMB, and 70PP/30PET MFC at 210 °C.

Thermogravimetric analyses of the samples were performed to investigate the degradation of PP and PET in IMB and MFC compared with virgin PP and PET. The results are shown in Figure 3a and summarized in Table 1. All samples show a single weight loss upon heating to 600 °C. The degradation step of PP was within the range of 342 to 436 °C with a limited char yield of 0.1% and for PET within 396 to 491 °C with a char yield of 14.5%. Those results are very consistent with literature [9,13,33]. The thermal degradation of PP does not imply chain branching or crosslinking [33], so the process started by thermal scission of C–C chain bonds [34]. As can be expected, if we compare the decompositions of PP and PET, it is obvious that PET has a higher thermal stability than PP. PET’s decomposition is initiated by scission of the ester group in the chain, yielding to carboxyl and vinyl ester groups, and eventually 14.5% of char residue remains. As the IMB and MFC contain a high amount of PET (30 wt %) the difference between the PP degradation and the other two is obvious. A delay in the onset temperature for IMB and MFC due the presence of the PET phase can be noticed. The higher temperature onset for MFC indicates the delayed degradation due to the formation of PET microfibrils. From the first derivative (dTG) of thermogravimetric (TG) curves represented in Figure 3b, can be seen that the presence of PET severely affects the degradation behavior of PP in both IMB and MFC. The degradation peaks for IMB and MFC were found at 419.1 and 430.8 °C, respectively. Due to the presence of PET in the fibril shape, the degradation of the blend is strongly affected, as the fibrils are prone to degrade slower than particles. Even after melting, orientation and alignment of fibrils can be maintained, since only temperature is applied during the test (no shear or mixing) so the alignment remains in the molten phase, which results in larger cohesion forces and later onset of degradation. Jayanarayanan et al. [13] elaborated that a high aspect ratio of PET microfibrils (five or eight) can shift the temperature onset of decomposition in MFC.

Differential scanning calorimetry (DSC) was used for studying the melting and crystallization behavior of PP and PET in the IMB and MFC samples. Table 2 shows the results of thermal properties during first heating of all samples. The melting temperature (*T*_m_), crystallization temperatures (*T*_c_), *T*_c_^onset^, and *T*_c_^endset^, which indicate the temperature at the beginning and end of crystallization, heat of fusion ΔH_m_ of the PP phase and the PET phase, absolute value of crystallization enthalpy Δ*H*_c_ of PP, and percentage of crystallinity (α_c_) are displayed.

In Table 2 it can be seen that the melting enthalpy for IMB is lower than that of the pure PP, which results in a lower crystallinity degree in IMB. There is a possibility that mobility of PP chains was reduced due to the presence of the PET phase [6]. However, in the MFC, α_c_ is higher in comparison to both pure PP and IMB, due to the long fibrils. These act as nucleation sites for the trans-crystallization of the PP phase [13], thereby increasing the total amount of crystallinity. In IMB, this nucleation effect is much lower and dominated by the constraining effect of the PP phase, slightly reducing α_c_.

**Figure 4:**
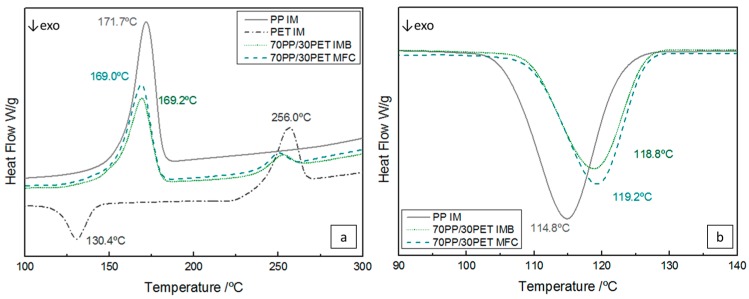
DSC thermograms of PP IM, PET IM, 70PP/30PET IMB, and 70PP/30PET MFC during (**a**) heating; and (**b**) cooling.

Figure 4a represents the DSC thermograms of the samples after injection molding. The temperature of 171.7 °C corresponds to the melting peak of the PP phase, during which time PET is still a crystalline solid, while the PP is in a high melting state. Analyzing the PET curve, a typical cold crystallization at 130.4 °C and the melting point at 256 °C were found. Comparing the T_m_ for IMB and MFC with pure PP, a decrease in temperature can be found, still with a single melting peak of PP, which confirms the presence of α-crystalline modification. The crystallization temperature of neat PP is around 114 °C, but in IMB the PET phase-shifted *T*_c_ of PP to the higher levels ~120 °C (Figure 4b). PP crystals become imperfect due to the presence of the PET phase, which means a lower spherulite size of PP and a higher number of spherulites. Therefore, the result is a decrease of the melting temperature of PP and an enhanced crystallization. In the case of MFC the same effect is noted, which indicates that PET in situ microfibers had a significant nucleating effect on the crystallization of the PP phase due to the higher surface-to-volume ratio. Similar results have been described by other authors [6,8,13,35,36].

### 3.2. Morphology of the IMBs and MFCs

#### 3.2.1. Morphology Development

**Figure 5:**
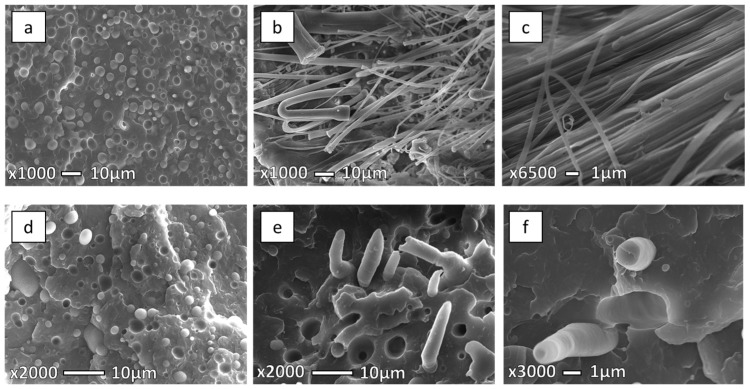
SEM micrographs of freeze-fracture surfaces under liquid nitrogen of the 70PP/30PET (**a**) extrusion blend; (**b**,**c**) stretched blend; (**d**) IMB at 210 °C; (**e**,**f**) MFC at 210 °C.

Morphological control of the dispersed phase is extremely important to achieve good properties for polymer blends and microfibrillar composites [2,4,9,25,37,38]. Figure 5 represents SEM micrographs of PP/PET composition with 30 wt % PET after extrusion, stretching, and injection molding at 210 °C. The unstretched blend shows a typical incompatible morphology (Figure 5a), where the spherical PET particles with a diameter ranging between 1–5 μm are found to be distributed in the PP matrix. Additionally, it can be seen that the PET particles exhibit a uniform dispersion in the PP matrix, with no adhesion between the two phases, which indicates a completely immiscible blend [2,4]. This morphology is very convenient for stretching and making fibers, and later, MFCs.

Figure 5b,c show the morphology after fibrillation and before injection molding. It is clear that both phases are oriented and the average diameter of the fibrils is 1.5 μm. Figure 5d presents the IMB sample injection molded at 210 °C with obvious incompatible blend morphology. Discrete domains of the minor phase dispersed within the continuous phase of the major phase without adhesion between the phases can be seen. After injection molding at 210 °C it can be noticed that the MFC structure obtained after stretching was preserved in the samples (Figure 5e,f). Fibers with diameters of 1–2 μm can be found.

#### 3.2.2. Influence of *T*_im_ on Morphology

**Figure 6:**
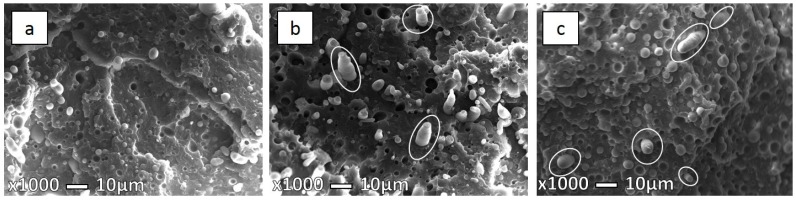
SEM micrographs of freeze-fracture surface under liquid nitrogen 70PP/30PET IMB at (**a**) 210 °C; (**b**) 230 °C; and (**c**) 280 °C.

IMBs and MFCs were injection molded at three different injection molding temperatures of 210, 230, and 280 °C. Figure 6 shows the different morphologies of the IMBs at different *T*_im_. All prepared IMBs and MFCs, either low or high *T*_im_ show typical incompatible morphology. At 230 and 280 °C (Figure 6b,c), some larger elliptical particles occur, which could be mistaken for microfibrils, but contrary to microfibrils they have uneven cross-sections. Several PET particles have made new single daughter particles, ellipsoids which, from the obtained observation, could be mistaken for fibrils, but it is known that blend morphology does not display these structures. This phenomenon is known as a coalescence effect. Under the effect of coalescence, the merging of two or more particles into one new larger particle which is often not spherical in shape is implied [25]. During the injection molding process at higher temperature there is a significant possibility for deformation and coalescence of PET particles and fibrils [13,14,24,25,30,38,39,40,41,42,43,44,45].

In Table 3 the average, minimum, and maximum diameters of PET particles and fibers in PP/PET compositions are listed. Comparing the size of PET particles at lower and higher *T*_im_, the particles at lower *T*_im_ are smaller (1–2.5 μm), while the particle size at 280 °C can reach diameters up to 6 μm due to the aforementioned coalescence.

Higgins et al. [19] explained that is difficult to control the mixture of polymers as they are thermodynamically stable only in limited temperature ranges. As a consequence, the uncompatibilized blends at high processing temperature will show larger-sized domains, as was confirmed by Van Bruggen et al. in a recent study [46].

**Figure 7:**
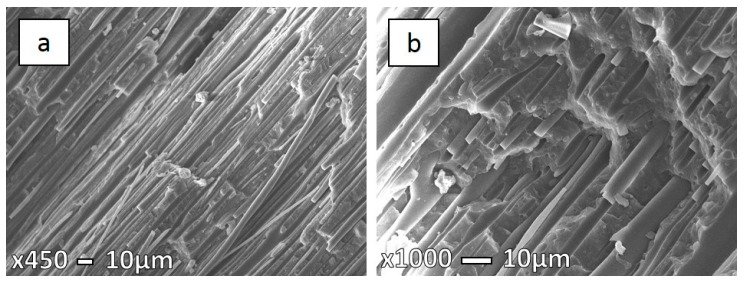
SEM micrographs of freeze-fracture surfaces in the parallel direction under liquid nitrogen of 70PP/30PET MFC at 210 °C (**a**) low magnification (×450); and (**b**) high magnification (×1000).

**Figure 8:**
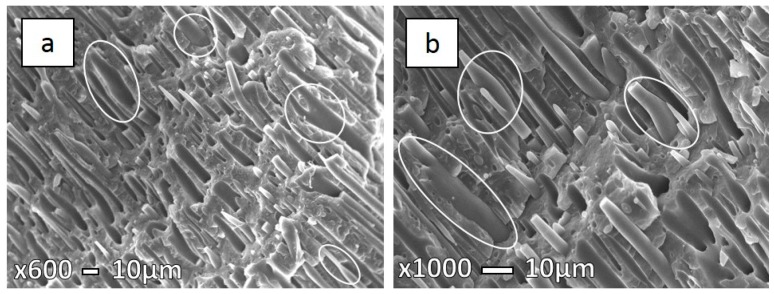
SEM micrographs of freeze-fracture surfaces in the parallel direction under liquid nitrogen of 70PP/30PET MFC at 230 °C (**a**) low magnification (×600); and (**b**) high magnification (×1000).

**Figure 9:**
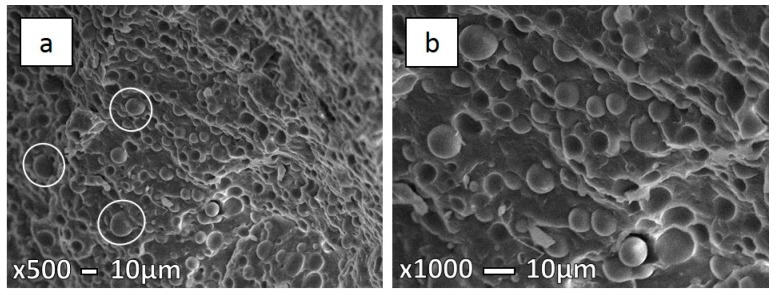
SEM micrographs of freeze-fracture surfaces in the parallel direction under liquid nitrogen of 70PP/30PET MFC at 280 °C (**a**) low magnification (×500); and (**b**) high magnification (×1000).

Micrographs presented in the Figure 7, Figure 8 and Figure 9 show the specimen fracture of MFC in the parallel direction to injection molding flow. It was hard to measure the exact longitude of fibers from these micrographs as the matrix was not removed, so some fibers are hidden, while the diameters were measured (Table 3). As mentioned earlier above, coalescence has a significant influence for PET fibers due to low PET viscosity. Hence, a significant possibility is that long fibers deform and, afterwards, stick together. This is more likely in the case of higher processing temperatures, but we cannot exclude that it could happen during lower processing temperatures. The obtained microstructure of MFC made at 210 °C (Figure 7a) clearly indicates the uniformity in fibers, as well their size. On the other hand, in the case of the sample at 230 °C (Figure 8) a larger variation in fiber diameter can be observed, as well as some necking through the length of the fibers.

Körmendy et al. [24] explained that the particles and fibers break up and their coalescence is supported by their amorphous state in the PP matrix. The reason for the larger fiber diameter in the case of MFCs made at 230 °C is probably caused by the high shear stress at elevated temperatures during injection molding. Long fibrils cannot withstand this, so they are prone to deform, break up, and coalesce [13,25]. At elevated temperatures, such as 280 °C, PET fibers are effectively re-melted and yield a different morphology altogether, as can be seen in Figure 9. The obtained microstructure once more resembles the IMB.

Definitely, the control of *T*_im_ is of great importance during this step. We have seen that there is an extreme possibility that PET particles and fibers are broken in IMBs and MFCs. From our point of view, the long PET fibers made during cold stretching at high temperature are lost, as the PET was melted again at its processing temperature, which is also confirmed for the IMB sample. Similar explanations were found in several studies [14,38,47].

### 3.3. Mechanical Properties

#### 3.3.1. Development of Mechanical Properties

Generally, it is known that uncompatibilized blends have inferior mechanical properties compared with the virgin polymers [15]. In order to achieve superior properties and improve them in IMBs and MFCs, our focus was to investigate the influence of *T*_im_ on those properties.

In Table 4 the results obtained for samples made at 210 °C are listed. The impact energy for both PP/PET IMB and MFC was lower than for neat PP and PET. This is due to the incompatibility between PP and PET and the occurrence of phase separation [6,38]. MFC showed a slight increase of 8% in comparison with IMB (*p* = 0.057) for the impact energy. The reason of the enhanced impact properties in MFC, as explained by some authors [17,29,48], probably lies in the presence of PET fibers which are more resistant to crack propagation. Perkins et al. [48] explained that impact resistance can vary depending on the degree of crystallinity. In our case PET fibers reinforce the individual PP spherulites. As it has been mentioned earlier, the PP crystals become imperfect and smaller, which may increase the impact strength. Both the size and amount of PP spherulites play an important role and, together with the interfacial adhesion between the oriented PET fibers and the PP matrix, will determine the impact strength.

Comparing the flexural modulus of IMB and MFC with that of pure PP, a significant difference was found: an increase of 37% (*p* = 0.000) and 42% (*p* = 0.000) is noted, respectively. IMB (*p* = 0.00) showed a significant increase in flexural strength relative to PP (*p* = 0.000) of 22%, while MFC (*p* = 0.001) increased a smaller amount, up to 12%.

Due to the high flexural properties of PET, the IMB and MFC have shown an increase in flexural modulus and strength in relation to neat PP. Comparing the flexural modulus of IMB to MFC (*p* = 0.011), a small, yet significant, improvement was observed, while flexural strength was significantly higher for IMB compared with MFC (*p* = 0.002). Confirmations are found by other researchers, as well [3,26,35].

#### 3.3.2. Influence of *T*_im_ on Mechanical Properties

**Figure 10:**
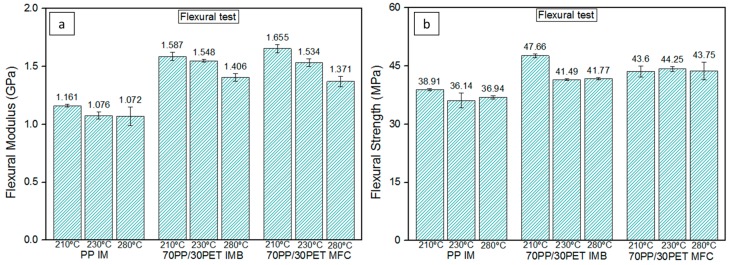
Comparison of flexural properties of PP IM, 70PP/30PET IMBs, and 70PP/30PET MFCs at three different *T*_im_ (**a**) flexural modulus; (**b**) flexural strength.

Figure 10 represents a comparison between the flexural properties at different *T*_im_. The mechanical properties of the PP samples at different temperatures are in the same range. However, there is a significant difference between 210 °C and the high temperature of 280 °C (*p* = 0.00) in flexural modulus, while no significant difference between 230 and 280 °C is found (*p* = 0.779), as well as between 210 and 230 °C (*p* = 0.779). Furthermore, there is no difference in flexural strength between 230 and 280 °C (*p* = 0.399), and between 210 and 230 °C (*p* = 0.399). On the other hand, there is a significant difference between 210 and 280 °C. The differences probably originate from different skin-core structures. Several researchers studied the formation of skin-core structures of IM parts. Yi et al. [49], for one, described how, during injection, the skin layer is exposed to a high stress, strain, and large cooling rate, all of which lead to different morphology than in the core. This skin-core structure is, amongst others, influenced by melt temperature and hold pressure elaborated by Zhou et al. [50].

There is an evident increase in the flexural properties for IMB and MFC injection molded at 210 and 230 °C relative to the samples made at 280 °C. The IMB samples processed at lower *T*_im_ showed an increase in flexural modulus in comparison with 280 °C (Figure 10a). However, for the flexural strength of IMB was found a significant increase in the case of 210 °C relative to 230 and 280 °C (*p* = 0.00) (Figure 10b).

Furthermore, the MFC samples made at 210 °C showed a significant improvement for the flexural modulus compared to both 230 and 280 °C (*p* = 0.00). On the other hand, there are no differences found for flexural strength at different *T*_im_. Comparing the flexural strength of IMB and MFC at 210 °C, IMB showed a superior value. It could be explained that the strain at maximum stress, **e**_FSmax_, is higher for IMB (Table 4). As straining is allowed to continue further than for MFC, it makes sense that higher stress levels are also reached. The higher **e**_FSmax_ for IMB is due to the fact that the spherical PET parts are in a previously unstretched state, as opposed to the PET microfibrils in MFC, which have undergone strain hardening during the stretching. This will lead to a reduced ductility of the microfibrils.

The PP impact results between different *T*_im_ show the slight variations. This is, again, due to the different skin-core structure and PP crystal morphology, strongly affected by the melt temperature [50]. Perkins et al. [48] reported that the combination of high melting temperature, low mold temperature, and fast injection time will give the optimal impact strength.

The impact energy for the IMB samples at the lowest *T*_im_ has the highest value. This is probably due to the smaller particle size. According to the measured diameters listed in Table 3, there is no significant difference in the average particle size for IMB samples at 210 and 230 °C but, looking into the morphology, there was found a more uniform dispersion of the small PET particles in the case of 210 °C, while the sample at 230 °C shows a distribution of the higher particle diameter. Moreover, in Figure 6b, some collapsed elliptical particles are noticeable which can reduce the final mechanical properties.

In the case of the MFC sample, we found a significant difference between 210 and 280 °C (*p* = 0.022) and, as it can be seen from the graph, there are no differences between 230 and 280 °C (*p* = 0.55).

**Figure 11:**
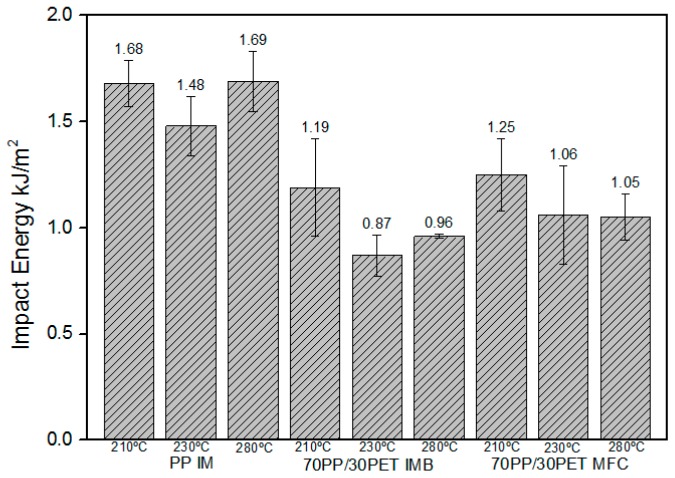
Comparison of impact energy (kJ/mm) of PP IM, 70PP/30PET IMBs, and 70PP/30PET MFCs at three different *T*_im_.

However, the impact energy for MFC made at 210 °C is higher than that made at 280 °C, as we expected. There is a strong possibility that long PET microfibrils can act as nucleating agents for the PP, which will lower the PP crystal size and improve the adhesion between the two phases [29]. The MFC made at 280 °C has converted into a blend, as was confirmed by its morphological development, and its value corresponds to that of IMB. Comparing all obtained values for impact strength at lower and higher *T*_im_, IMBs and MFCs at high temperature have shown inferior impact properties (Figure 11).

According to Friedrich et al. [3] and Jayanarayanan et al. [29] processing by injection molding has a great influence on PET particles and fibrils. Summarizing all of the results from Figure 10 and Figure 11, the differences between the samples processed at low and high temperatures are obvious. The samples produced at lower temperatures have shown better properties, but also improvements in properties were shown when dealing with the MFC structure over IMB.

At high *T*_im_, the MFCs lose the morphology obtained by twin-screw extrusion and cold stretching, due to the coalescence of PET fibers, which will affect the mechanical properties. During injection molding the PET fibers are exposed to high shear stresses and relaxation behavior at elevated temperatures, which causes the reduction of their aspect ratio and the decrease in properties. As it was shown, for both IMB and MFC samples made at 280 °C, the lowest properties were obtained, which could be explained by a fact that, at the high temperature, PET particles, as well fibers, were re-melted again, losing the original morphology. In addition, the explanation could lie in the effect of coalescence. At the higher temperature, the PET phase is in a molten state and, according to their microstructures, it is evident that PET fibers (particles) were deformed and broken which, afterwards, during mixing, resulted in their interaction and coalescence (see Section 3.2.2). It is known that reinforcing fibers will only act as an effective stress transfer agent up to a certain critical length, which is related to the adhesion between the matrix and the fibers [29], so they do have a tendency to break into shorter ones. This fiber breakup will affect the mechanical strength, as was shown in the results, and confirmed by a morphology study.

We have shown that *T*_im_ strongly affects the mechanical properties of all samples, especially MFC, as they lose their fibril morphology at the highest processing temperature. In terms of maximizing the mechanical properties, these could be obtained by mitigating the immiscibility between PP and PET [17,21,26,28,38,47] or increasing the draw ratio [26,29] (which was deliberately kept low within the current work).

It was, however, not the purpose of this manuscript to maximize mechanical properties, but to investigate the separate effect of *T*_im_ on morphology and other properties. A combination of these insights with the aforementioned known strategies to increase mechanical properties would potentially create a synergistic optimizing effect.

In this, it is very important to maintain high adhesion between the two phases and a high aspect ratio of reinforcement [25] to achieve superior mechanical properties. This could be achieved beside a good control of injection molding temperature and other settings during processing by, for example, adding a compatibilizer [7,26,28,38,47], using chemical interactions [3,51] and transcrystallinity phenomenon [3,25,27,52], and controlling the settings during cold stretching and injection molding.

## 4. Conclusions

The influence of injection molding temperature on morphology and properties of IMBs and MFCs based on PP and PET was studied. Unstretched blends and microfibrillar composites of PP/PET (70/30, *w/w*) were successfully achieved by two (extrusion/injection molding) or three processing steps (extrusion/cold stretching/injection molding), respectively.

SEM microscopy confirmed the immiscibility of PP and PET in all of the samples, as well as the existence of highly oriented PET microfibrils after cold stretching. Moreover, after injection molding at low *T*_im_ the MFC structure with oriented fibrils preserved its original morphology. At the highest *T*_im_, PET fibers were re-melted and a new morphology was obtained, much like the corresponding IMB. The phenomenon of coalescence was present in all IMBs and MFCs at high *T*_im_ as the diameter of particles and fibers was significantly higher, which indicates that the PET phase was effectively re-melted.

The crystallization temperature of PP phase in PP/PET IMBs and MFCs was shifted to higher temperatures around 120 °C, as PP crystals became imperfect due to the presence of the PET phase. The crystallinity degree in the MFC was found to be higher than in both neat PP and IMB, as the PET fibrils act as nucleating agents for the transcrystallization of PP and dominates the constraining effect. Additionally, due to the existence of the PET phase in IMBs and MFCs, the poor thermal stability of PP was improved. Analysis of the degradation peaks via dTG curves confirmed the later temperature onset for the MFC sample, due to the formation of PET fibrils, which are harder to decompose then spheres.

The mechanical tests have confirmed that the morphology and properties are significantly affected by processing temperature. The flexural modulus and strength of the IMBs and MFCs were found to be superior to those of neat PP. MFCs have shown a greater impact strength in relation to IMBs, which confirms that PET fibers are a tough reinforcement for the weak PP/PET blend. Both IMB and MFC at elevated *T*_im_ (280 °C) have shown inferior properties, since the materials have lost their original morphology and the PET phase was re-melted, accompanied by coalescence. The rather limited improvement in mechanical properties of the MFC is attributed to the low draw ratio and low adhesion between the matrix and reinforcement.

Finally, on the basis of these results, it can be concluded that the control of temperature during processing of MFCs is of huge importance, in the same way that the other known factors, such as control of the size of the dispersed phase, draw ratio, and adhesion between the matrix and reinforcement are.

## Figures and Tables

**Table 1:** Non-isothermal decomposition characteristics of PP IM, PET IM, 70PP/30PET IMB, and MFC in nitrogen.

Sample	*T*^onset^ (°C)	*T*^max^ (°C)	*T*^endset^ (°C)	Char yield at 550 °C (wt %)
PP IM	342.1	417.6	435.9	0.1
PET IM	396.5	441.4	490.8	14.5
70PP/30PET IMB	349.3	419.1	469.2	4.7
70PP/30PET MFC	361.5	430.8	470.2	5.8

**Table 2:** Thermal properties of PP IM, PET IM, 70PP/30PET IMB, and MFC during the first heating and cooling.

System	Heating						Cooling	
Sample	*T*_m_^PP^ (°C)	Δ*H*_m_^pp^ (J/g)	*T*_m_^PET^ (°C)	Δ*H*_m_^PET^ (J/g)	α_c_^PP^ %	*T*_c_^PP^ (°C)	*H*_c_^PP^ (J/g)	*T*_c_^PET^ (°C)
PP IM	171.7	73.74	-	-	35.6	114.8	112.80	-
PET IM	-	-	256.0	30.13	-	-	-	196.3
70PP/30PET IMB	169.2	45.60	254.9	5.85	31.5	118.8	72.12	189.1
70PP/30PET MFC	169.0	55.89	250.7	5.37	38.6	119.2	77.84	193.0

**Table 3:** Diameters of PET spherical particles and fibers in PP/PET composition.

Sample	Temperature (°C)	Diameter min/max fiber or sphere (μm)	Average diameter (μm)
Extrusion blend	-	1.0–5.0	2.8 ± 1.25
Stretched blend	-	0.5–2.0	1.5 ± 1.81
70PP/30PET IMB	210	1.0–2.5	1.7 ± 0.72
	230	1.0–3.0	2.0 ± 0.79
	280	2.0–6.0	3.6 ± 1.21
70PP/30PET MFC	210	1.0–2.0	1.8 ± 1.04
	230	2.5–6.5	3.7 ± 1.47
	280	3.0–7.2	4.2 ± 1.80

**Table 4:** Impact and flexural properties of PP IM, PET IM, 70PP/30PET IMB, and MFC at 210 °C.

Sample	Samples made at 210 °C			
	Impact	Flexural		
	Strength (kJ/m^2^)	Modulus (GPa)	Strength (MPa)	Strain at maximum flexural stress (%)
PP IM	1.68 ± 0.11	1.161 ± 0.010	38.91 ± 0.29	8.11 ± 0.13
PET IM	2.32 ± 0.39	2.301 ± 0.056	81.95 ± 1.48	5.68 ± 0.09
70PP/30PET IMB	1.19 ± 0.22	1.587 ± 0.034	47.66 ± 0.45	5.82 ± 0.11
70PP/30PET MFC	1.25 ± 0.17	1.655 ± 0.030	43.60 ± 0.03	5.50 ± 0.28

## References

[1] Li J., Yu X., Guo S. (2007). Development of morphology and properties of injection-molded bars of HDPE/PA6 blends. J. Polym. Sci. Part B Polym. Phys..

[2] Li Z.M., Yang W., Xie B.H., Shen K.Z., Huang R., Yang M.B. (2004). Morphology and tensile strength prediction of in situ microfibrillar poly(ethylene terephthalate)/polyethylene blends fabricated via slit-die extrusion-hot stretching-quenching. Macromol. Mater. Eng..

[3] Friedrich K., Evstatiev M., Fakirov S., Evstatieva O., Ishiic M., Harrassa M. (2005). Microfibrillar reinforced composites from PET/PP blends: Processing, morphology and mechanical properties. Compos. Sci. Technol..

[4] Xu L., Zhong G.J., Ji X., Li Z.M. (2001). Crystallization behaviour and morphology of one-step reaction compatibilized microfibrillar reinforced isotactic polypropylene/poly(ethylene therephthalate) (iPP/PET) blends. Chin. J. Polym. Sci..

[5] Shields R.J., Bhattacharyya D., Fakirov S. (2008). Oxygen permeability analysis of microfibril reinforced composites from PE/PET blends. Compos. Part A Appl. Sci. Manuf..

[6] Mirjalili F., Moradian S., Ameri F. (2013). Enhancing the dyeability of polypropylene fibers by melt blending with polyethylene terephthalate. Sci. World J..

[7] Canetti M., Bertini F. (2007). Supermolecular structure and thermal properties of poly(ethylene terephthalate)/lignin composites. Compos. Sci. Technol..

[8] Ujhelyiová A., Bolhová E., Marcinčin A., Tiňo R. (2007). Blended polypropylene/polyethylene terephthalate fibres: Crystallisation behaviour of polypropylene and mechanical properties. Fibres Text. East. Eur..

[9] Inuwa I.M., Hassan A., Samsudin S.A., Mohamad Haafiz M.K., Jawaid M. (2015). Interface modification of compatibilized polyethylene terephthalate/polypropylene blends: Effect of compatibilization on thermomechanical properties and thermal stability. J. Vinyl Addit. Technol..

[10] Shields R.J., Bhattacharyya D., Fakirov S. (2008). Fibrillar polymer–polymer composites: Morphology, properties and applications. J. Mater. Sci..

[11] Perez L.A., Rodriguez D.N., Rodriguez F.J., Hsiao B., Avila-Orta C.A., Sics I. (2014). Molecular weight and crystallization temperature effects on poly(ethylene terephthalate) (pet) homopolymers, an isothermal crystallization Analysis. Polymers.

[12] Kayaisang S., Saikrasun S., Amornsakchai T. (2013). Potential use of recycled pet in comparison with liquid crystalline polyester as a dual functional additive for enhancing heat stability and reinforcement for high density polyethylene composite fibers. J. Polym. Environ..

[13] Jayanarayanan K., Thomas S., Joseph K. (2011). In situ microfibrillar blends and composites of polypropylene and poly (ethylene terephthalate): Morphology and thermal properties. J. Polym. Res..

[14] Li Z.M., Yang M.B., Feng J.M., Yang W., Huang R. (2002). Morphology of in situ poly(ethylene terephthalate)/polyethylene microfiber reinforced composite formed via slit-die extrusion and hot-stretching. Mater. Res. Bull..

[15] Chiu H.T., Hsiao Y.K. (2006). Compatibilization of Poly(ethylene terephthalate)/Polypropylene Blends with Maleic Anhydride Grafted Polyethylene-Octene Elastomer. J. Polym. Res..

[16] Li Z.M., Li L., Shen K.Z., Yang M.B., Huang R. (2005). In situ poly(ethylene terephthalate) microfibers- and shear-induced non-isothermal crystallization of isotactic polypropylene by on-line small angle X-ray scattering. Polymer.

[17] Asgari M., Masoomi M. (2012). Thermal and impact study of PP/PET fibre composites compatibilized with Glycidyl Methacrylate and Maleic Anhydride. Compos. Part B Eng..

[18] Laukaitiné A., Jankauskaité V., Žukiené K., Norvydas V., Munassipov S., Janakhmetov U. (2013). Investigation of polyvinyl chloride and thermoplastic polyurethane waste blend miscibility. Mater. Sci..

[19] Higgins J.S., Lipson J.E.G., White R.P. (2010). A simple approach to polymer mixture miscibility. Philos. Trans. R. Soc. A.

[20] Jiang W., Du M., Gu Q., Jiang J., Huth H., Zhou D., Xue G., Schick C. (2010). Calorimetric study of blend miscibility of polymers confined in ultra-thin films. Eur. Phys. J. Spec. Top..

[21] Champagne M.F., Huneault M.A., Roux C., Peyrel W. (1999). Reactive compatibilization of polypropylene/polyethylene terephthalate blends. Polym. Eng. Sci..

[22] Fakirov S., Bhattacharyya D., Shields R.J. (2008). Nanofibril reinforced composites from polymer blends. Colloids Surf. A.

[23] Fuchs C., Bhattacharyya D., Fakirov S. (2006). Microfibril reinforced polymer–polymer composites: Application of Tsai-Hill equation to PP/PET composites. Compos. Sci. Technol..

[24] Körmendy E., Marcinčin A., Hricová M., Kovačic V. (2005). Phase Morphology of Polypropylene-Polyethylene Terephthalate Blend Fibres. Fibres Text. East. Eur..

[25] Fakirov S., Bhattacharyya D., Lin R.J.T., Fuchs C., Friedrich K. (2007). Contribution of coalescence to microfibril formation in polymer blends during cold drawing. J. Macromol. Sci. Phys..

[26] Evstatiev O., Evstatiev M., Friedrich K. (2005). Effect of Compatibilization on the Properties of Microfibrillar Reinforced Composites Based on Poly(ethyleneterephthalate) and Polypropylene.

[27] Friedrich K., Ueda E., Kamo H., Evstatiev M., Krasteva B., Fakirov S. (2002). Direct electron microscopic observation of transcrystalline layers in microfibrillar reinforced polymer-polymer composites. J. Mater. Sci..

[28] Li W. (2009). PET/PP-Based Polymer Composites: Effects of Compatibilizer and Nanofillers on the Processing-Structure-Property Relationships. Ph.D. Thesis.

[29] Shibata S., Bozlur R.M., Fukumoto I., Kanda Y. (2010). Effects of injection temperature on mechanical properties of bagasse/polypropylene injection molding composites. BioResources.

[30] Jayanarayanan K., Joseph K., Thomas S., Bhattacharyya D., Fakirov S. (2012). Microfibrils Reinforced composites based on PP and PET: Effect of draw ratio on morphology, static and dynamic mechanical properties, crystallization and rheology. Synthetic Polymer-Polymer Composites.

[31] Viana J.C., Alves N.M., Mano J.F. (2004). Morphology and mechanical properties of injection molded poly(ethylene terephthalate). Polym. Eng. Sci..

[32] Jayanarayanan K., Bhagawan S.S., Thomas S., Joseph K. (2008). Morphology development and non-isothermal crystallization behaviour of drawn blends and microfibrillar composites from PP and PET. Polym. Bull..

[33] Peterson J.D., Vyazovkin S., Wight C.A. (2001). Kinetics of the thermal and thermo-oxidative degradation of polystyrene, polyethylene and poly(propylene). Macromol. Chem. Phys..

[34] Kashiwagi T., Grulke E., Hilding J., Harris R., Awad W., Douglas J. (2002). Thermal degradation and flammability properties of poly(propylene)/carbon nanotube composites. Macromol. Rapid Commun..

[35] Zhu Y., Liang C., Bo Y., Xu S. (2015). Non-isothermal crystallization behavior of compatibilized polypropylene/recycled polyethylene terephthalate blends. J. Therm. Anal. Calorim..

[36] Thanomchat S., Srikulkit K., Suksut B., Schlarb A.K. (2014). Morphology and Crystallization of Polypropylene/Microfibrillated Cellulose Composites. Int. J. Appl. Sci. Technol..

[37] Shields R.J., Bhattacharyya D., Fakirov S., Bhattacharyya D., Fakirov S. (2012). Application opportunities of the microfibril reinforced composite concept. Synthetic Polymer-Polymer Composites.

[38] Lei Y., Wua Q., Zhang Q. (2009). Morphology and properties of microfibrillar composites based on recycled poly(ethylene terephthalate) and high density polyethylene. Compos. Part A Appl. Sci. Manuf..

[39] Jayanarayanan K., George G., Thomas S., Joseph K. (2009). Morphology development of normal blends, microfibrillar blends and composites from LDPE and PET. Acad. Rev..

[40] Perilla J.E., Jana S.C. (2005). Coalescence of immiscible polymer blends in chaotic mixers. AIChE J..

[41] Huang W., Shen J., Chen X. (2003). Effect of composition on phase morphology and mechanical properties of PP/PA66 in situ composites via extrusion-drawing-injection method. J. Mater. Sci..

[42] Padilla-Lopez H., Vazquez M.O., González-Núñez R., Rodrigue D. (2003). Influence of postextrusion parameters on the final morphology of polystyrene/high density polyethylene blends. Polym. Eng. Sci..

[43] Rodriguez-Gonzalez F.J., Virgilio N., Ramsay B.A., Favis B.D. (2003). Influence of melt drawing on the morphology of one- and two-step processed LDPE/thermoplastic starch blends. Adv. Polym. Technol..

[44] Perilla J.E., Jana S.C. (2004). A time-scale approach for analysis of coalescence in processing flows. Polym. Eng. Sci..

[45] Andrzejewski J., Szostak M., Barczewski M., Krasucki J., Sterzynski T. (2014). Fabrication of the Self-Reinforced Composites Using Co-Extrusion Technique. J. Appl. Polym. Sci..

[46] Van Bruggen E.P.A., Koster R.P., Picken S.J., Ragaert K. (2016). Influence of processing parameters and composition on the effective compatibilization of polypropylene–poly(ethylene terephthalate) blends. Int. Polym. Process..

[47] Abdullah M.Z., Pechstein L., Lin R.J.T., Bhattacharyya D., Bhatangar N., Srivatsan T.S. (2009). Behaviour of microfibrillar composite blends during product manufacturing. Processing and Fabrication of Advanced Materials-XVI.

[48] Perkins W.G. (1999). Polymer toughness and impact resistance. Polym. Eng. Sci..

[49] Yi X., Chen C., Zhong G.J., Xu L., Tang J.H., Ji X., Li Z.M. (2011). Suppressing the skin–core structure of injection-molded isotactic polypropylene via combination of an in situ microfibrillar network and an interfacial compatibilizer. J. Phys. Chem. B.

[50] Zhou Y., Mallick P.K. (2005). Effects of melt temperature and hold pressure on the tensile and fatigue properties of an injection molded talc-filled polypropylene. Polym. Eng. Sci..

[51] Fakirov S., Fakirov S. (2008). Transreactions in Condensation Polymers.

[52] Krumova M., Michler G.H., Evstatiev M., Friedrich K., Stribeck N., Fakirov S. (2005). Transcrystallisation with reorientation of polypropylene in drawn PET/PP and PA66/PP blends. Part 2. Electron microscopic observations on the PET/PP blend. Progr. Colloid Polym. Sci..

